# Genome-wide-scale prediction of compound−protein interactions using foundation and language models based on three-dimensional structures of compounds and proteins

**DOI:** 10.1093/bioinformatics/btag710

**Published:** 2026-09-28

**Authors:** Yuga Moriyama, Yuki Matsukiyo, Tsuyoshi Kimura, Hiyori Yamamoto, Yuko Sakajiri, Tomokazu Shibata, Ryusuke Sawada, Yoshihiro Yamanishi

**Affiliations:** Department of Complex Systems Science, Graduate School of Informatics, Nagoya University, Nagoya, Aichi 464-8601, Japan; Department of Complex Systems Science, Graduate School of Informatics, Nagoya University, Nagoya, Aichi 464-8601, Japan; Department of Complex Systems Science, Graduate School of Informatics, Nagoya University, Nagoya, Aichi 464-8601, Japan; Department of Complex Systems Science, Graduate School of Informatics, Nagoya University, Nagoya, Aichi 464-8601, Japan; Department of Complex Systems Science, Graduate School of Informatics, Nagoya University, Nagoya, Aichi 464-8601, Japan; Department of Complex Systems Science, Graduate School of Informatics, Nagoya University, Nagoya, Aichi 464-8601, Japan; Integrated Glyco-Big Data Center (iGDATA), Institute for Glyco-core Research (iGCORE), Nagoya University, Nagoya, Aichi 464-8601, Japan; Department of Complex Systems Science, Graduate School of Informatics, Nagoya University, Nagoya, Aichi 464-8601, Japan; Division of Interdisciplinary Research and Development (R&D), Aichi Cancer Center Research Institute, Nagoya, Aichi 464-8681, Japan

## Abstract

**Motivation:**

The identification of compound–protein interactions (CPIs) is crucial in the early stages of drug discovery. However, machine-learning (ML)-based methods based on one- and two-dimensional representations cannot capture important geometric information on the binding sites of CPIs, which limits their predictive accuracy.

**Results:**

We propose DESTIN (dual structure-guided estimation of substance–target interaction networks), an ML framework for CPI prediction on a genome-wide scale from the three-dimensional (3D) structures of compounds and proteins. We constructed compound feature vectors that spatially reflect stable 3D conformations from a quantum–chemical perspective by fine-tuning a 3D structure-based foundation model. Furthermore, we constructed protein feature vectors leveraging sequence-based structure representations and protein language models, even for proteins that do not have 3D structures determined experimentally. CPI prediction was performed using an integrative ML model on 3D structure-aware feature vectors for compounds and proteins. The effectiveness of the proposed model was validated via rigorous benchmark tests on practical scenarios. DESTIN exhibited good predictive performance, even for structurally complex and pharmacologically important membrane proteins. These results show that DESTIN is promising for various pharmaceutical applications.

**Availability and Implementation:**

All source code and data supporting this study are publicly available on GitHub and archived on Zenodo (https://doi.org/10.5281/zenodo.21759701).

## 1 Introduction

Identification of compound–protein interactions (CPIs) is crucial in the early stages of drug discovery, particularly for screening bioactive compounds against target proteins and investigating off-target proteins (Keiser *et al.* 2009). Experimental approaches for CPI identification, such as in vitro assays, are costly and time-consuming; thus, there is an urgent need for more efficient computational screening methods. Various machine-learning (ML)-based CPI prediction methods have been proposed (Breiman, 2001; Nagamine and Sakakibara, 2007; Jacob and Vert, 2008; Yamanishi *et al.* 2008; Lee *et al.* 2019; Huang *et al.* 2021; Nguyen *et al.* 2021; Bai *et al.* 2023; Ma *et al.* 2024; Zhang *et al.* 2024; Zhao *et al.* 2024; Hadipour* et al.* 2025; Meng *et al.* 2025; Tian *et al.* 2025). These methods employ one-dimensional (1D) and two-dimensional (2D) representations, including SMILES strings, 2D graphs, molecular fingerprints for compounds, and amino acid sequences for proteins, as inputs. In reality, the interaction between a compound and a protein occurs in a three-dimensional (3D) space, where the spatial arrangement of atoms and the steric complementarity within the binding site play a crucial role in determining the interaction. Consequently, ML-based methods that rely solely on 1D and 2D representations cannot capture important geometric information about the binding sites, which limits their predictive accuracy.

3D molecular structures can be accurately modeled through quantum–chemical calculations (Cohen *et al.* 2012). For example, the conformations obtained from density functional theory (DFT) calculations provide physically reasonable structures; however, their high computational cost makes them unsuitable for large-scale datasets. As an alternative procedure, the 3D structural information of compounds can be efficiently incorporated using RDKit (Qiao *et al.* 2025). However, the conformations generated by RDKit may not reproduce the experimentally determined structures or conformations under physiological conditions—for example, they often result in nearly planar structures (Chen and Foloppe, 2013). Therefore, the reliability of 3D molecular representations remains a concern.

Traditional approaches to CPI analysis using 3D protein structures include molecular docking and molecular dynamics (MD) simulations (Hollingsworth and Dror, 2018; Sadybekov and Katritch, 2023). Molecular docking enables the prediction of binding affinities and poses between a compound and its target protein. MD simulations model the time-dependent motions of protein–ligand complex structures, providing insights into the dynamic nature of CPIs. However, these approaches are computationally expensive, limiting their application in large‑scale screening (Durrant and McCammon, 2011). In addition, they cannot be applied to proteins whose structures have not been experimentally determined.

In ML-based CPI prediction frameworks, the availability of 3D protein structure information is a major limitation. Although approximately 200 million natural proteins have amino acid sequences, only approximately 259,129 proteins have experimentally determined 3D structures (Service, 2020; Vallat *et al.* 2026). The recent advent of AlphaFold has enabled the direct acquisition of 3D protein structures from amino acid sequences (Jumper *et al.* 2021; Varadi *et al.* 2022). Information on 3D structures for arbitrary proteins is expected to be an important resource in drug discovery studies. Several studies have reported the successful identification of bioactive compounds using molecular docking based on protein structures predicted by AlphaFold (Lyu *et al.* 2024; Sawada *et al.* 2024). However, molecular docking is computationally expensive, making genome-wide CPI prediction impractical. Therefore, there is a need for ML-based CPI prediction frameworks that can effectively harness the 3D structural information on both compounds and proteins.

In this study, we propose DESTIN (dual structure-guided estimation of substance–target interaction networks), an ML framework that integrates the 3D structures of compounds and proteins to achieve high-accuracy CPI prediction on a genome-wide scale. For compounds, we considered the 3D coordinates of all atoms and pre-trained a 3D structure-based foundation model, constructing compound feature vectors that reflect spatially stable 3D conformations from a quantum–chemical perspective. For proteins, we leveraged sequence-based structure representations and protein language models enriched with 3D structure information to construct protein feature vectors, even for proteins that lack experimentally determined 3D structures. We conducted a rigorous benchmark study using multiple datasets and experimental settings, demonstrating the effectiveness of the proposed framework.

## 2 Methods

### 2.1 Overview of the proposed framework

In this study, we developed DESTIN, a framework that predicts CPIs using 3D structures of both compounds and proteins (Fig. 1).

**Figure 1:**
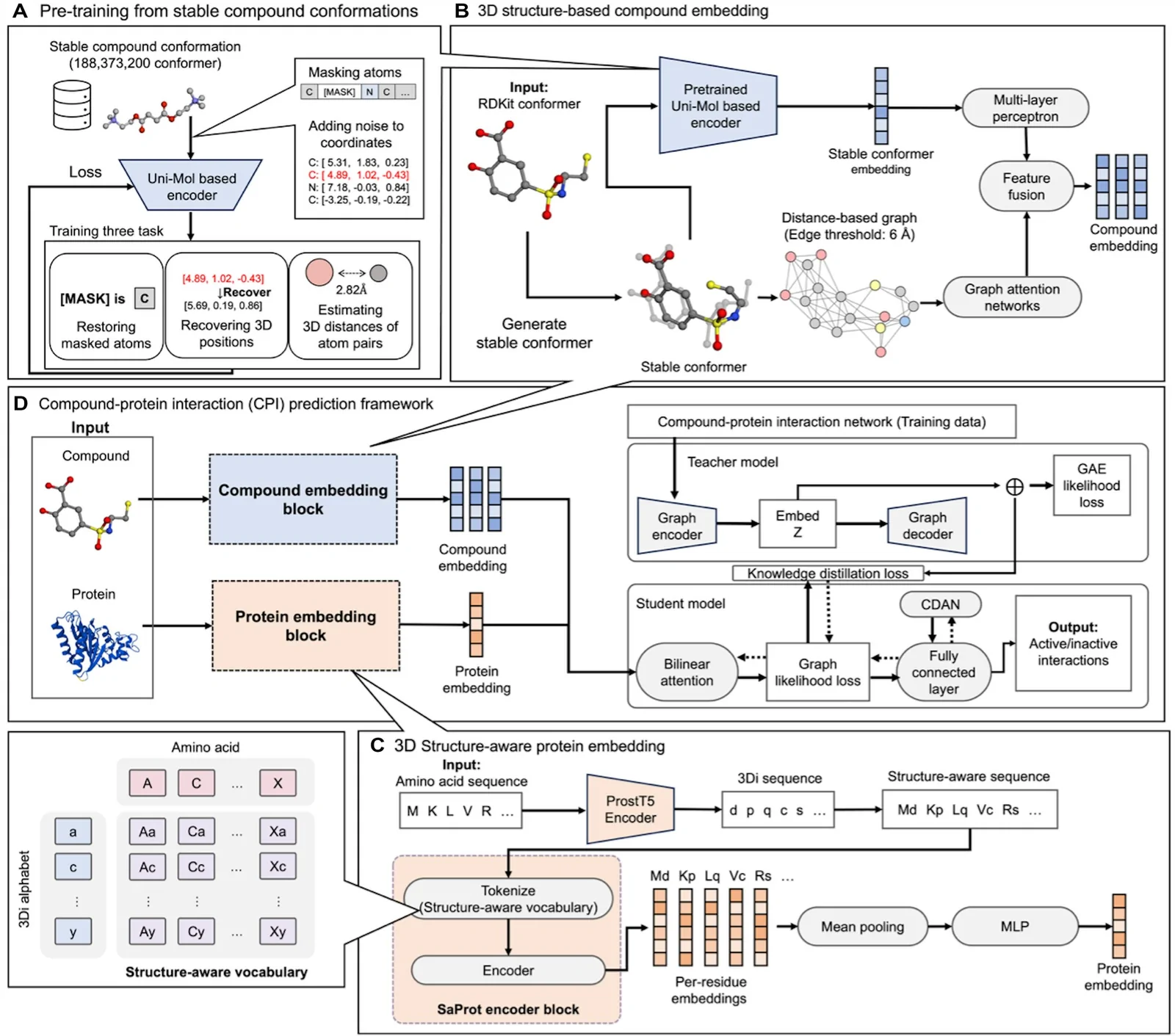
Overview of the DESTIN architecture. (A) Pretraining of Uni-Mol using structurally stabilized 3D compound structures. (B) Construction of compound feature vectors by integrating Uni-Mol- and graph-based embeddings, where edges are defined between atom pairs within 6 Å. (C) Construction of protein feature vectors by encoding structure-aware sequences with SaProt. The structure-aware sequences were constructed from amino acid and 3Di sequences predicted by ProstT5. (D) Knowledge distillation framework using a graph autoencoder as the teacher model. The student model consists of a BAN, fully connected layers, and a CDAN.

Figure 1A and B provides an overview of the construction of the compound feature. First, Uni-Mol (Zhou *et al.* 2023) is used to compute the embedding representations of the compounds. Uni-Mol is pretrained using structurally stabilized 3D compound structures (Fig. 1A). Next, the stabilized 3D structures are input into the pretrained Uni-Mol model to obtain compound embedding representations (Fig. 1B). These embeddings are reduced in dimension using a multi-layer perceptron. In parallel, the stabilized 3D compound structures are converted into distance-based graphs. The nodes in these graphs correspond to individual atoms. Edges are constructed between pairs of atoms with interatomic distances within 6 Å. The resulting graphs are input into graph attention networks (GAT) (Veličković *et al.* 2018). This process generates embedding representations from distance-based graphs. Finally, the Uni-Mol- and graph-based embeddings are combined to construct compound feature vectors.

Figure 1C shows the process of constructing protein feature vectors. First, amino acid sequences are input into ProstT5 (Heinzinger *et al.* 2024) to generate 3D interaction (3Di) sequences (van Kempen *et al.* 2024). Next, structure-aware sequences are constructed by alternately combining amino acids and 3Di alphabets. These structure-aware sequences are input into the pretrained SaProt model (Su *et al.* 2024). The output embeddings are used as protein feature vectors.

Figure 1D shows the overall CPI prediction framework. CPI prediction was performed using a knowledge distillation framework. A graph autoencoder (GAE) (Kipf and Welling, 2016) was used as the teacher model. The teacher model learns network information from a bipartite graph composed of compound–protein pairs in the training data and provides a knowledge distillation (KD) loss to the student model. The student model consists of a bilinear attention network (BAN), a conditional domain adversarial network (CDAN), and a classifier composed of fully connected layers. BAN fuses compound and protein feature vectors, and the classifier predicts the presence of interactions. During training, the student model is optimized to minimize the sum of losses from the teacher model, CDAN, and CPI prediction task.

### 2.2 Datasets

We used 4 benchmark datasets widely used in previous studies to evaluate the CPI prediction performance. The CPIs were retrieved from BindingDB (Gilson *et al.* 2016), BioSNAP (Zitnik *et al.* 2018), PDBbind 2016 (Cang *et al.* 2018), and KIBA (Tang *et al.* 2014). Table 1 lists the summary statistics of the datasets. Further details are provided in the “Dataset” section of the Supplementary Methods, available as supplementary data at *Bioinformatics* online.

**Table 1:** Summary statistics of the benchmark datasets used for compound–protein interaction (CPI) prediction.

Dataset	Compounds	Proteins	Active interactions	Inactive interactions
BindingDB	14,643	2,623	20,674	28,525
BioSNAP	4,505	2,181	13,830	13,634
PDBbind 2016	3,227	2,410	4,053	4,053
KIBA	2,068	229	22,729	95,525

### 2.3 Dataset splitting for performance evaluation

Two evaluation scenarios were employed to evaluate the predictive performance: the inductive and transductive scenarios. Figure 2 shows an overview of the 2 scenarios.

**Figure 2:**
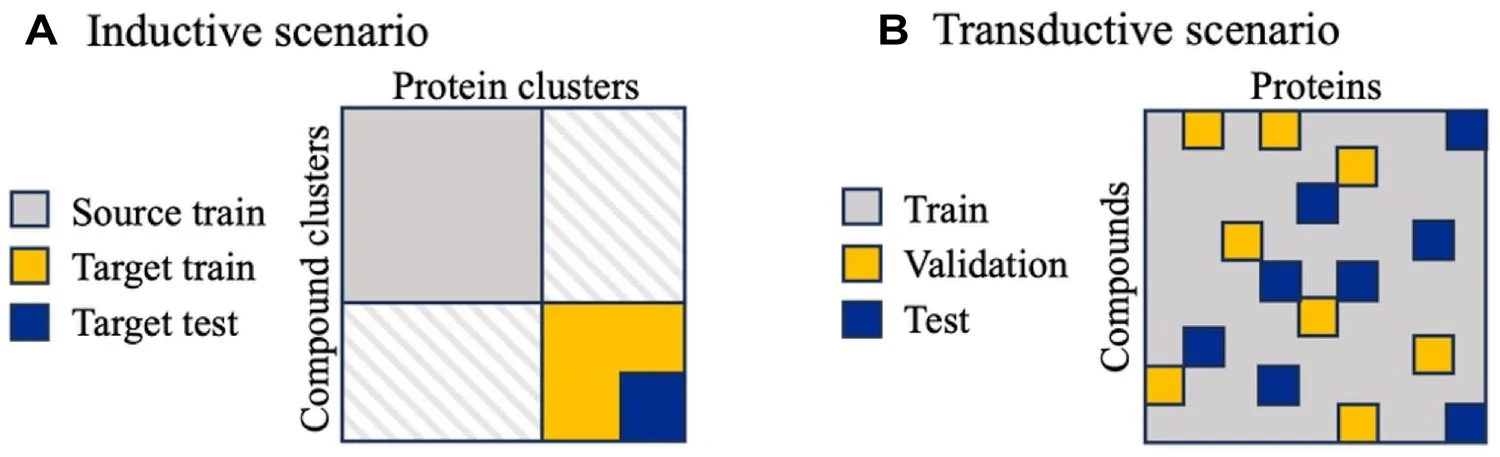
Overview of the dataset splitting scenarios for CPI prediction. (A) Inductive scenario. Compounds and proteins are clustered based on structural similarity, and data are split into source and target domains at the cluster level. The source domain (gray) contains only compound–protein pairs in which both the compound and protein clusters belong to the source domain. The target domain is further divided into target train (yellow) for domain adaptation and target test (navy blue) for evaluation. The hatched region indicates pairs in which only one of the compound or protein clusters belongs to the target domain, and these pairs are not used. (B) Transductive scenario. Compound–protein pairs are randomly divided into training, validation, and test sets. Compounds and proteins in the test set may also appear in the training set.

The inductive scenario reflects a more realistic situation than transductive scenario. In the inductive scenario (Fig. 2A), we evaluated the generalization performance for unseen compounds and proteins. Following the procedure of the performance evaluation on the state-of-the-art method (Hadipour *et al.* 2025), we performed clustering-based domain splitting. In the transductive scenario (Fig. 2B), we evaluated performance using random pair splitting. The entire dataset was randomly split into training (80%), validation (10%), and test (10%) sets. Details of the dataset splitting procedure are described in the “Dataset splitting for performance evaluation” section of the Supplementary Methods, available as supplementary data at *Bioinformatics* online.

### 2.4 Pretraining of 3D compound representations using structurally stabilized conformations

We generated compound embeddings derived from structurally stabilized conformations. We pretrained Uni-Mol (Zhou *et al.* 2023) using structurally stabilized 3D compound structures to compute embedding representations of compounds. Two large compound collections were used as pretraining data. The first collection contains approximately 12 million synthesizable and purchasable compounds provided by Uni-Mol, and the second collection contains approximately 11 million compounds collected from ZINC (Irwin and Shoichet, 2005) and ChEMBL (Gaulton *et al.* 2012). After removing duplicates, the resulting compounds were split into a training set of 18,837,320 compounds and a validation set of 98,844 compounds, which were used for pretraining. As a preprocessing step, structural stabilization was performed for compounds in the training and validation sets. The compounds were input into a Uni-Mol model fine-tuned on the GEOM-Drugs dataset (Axelrod and Gómez-Bombarelli 2022), and the resulting stabilized structures were used for subsequent training.

Uni-Mol was pretrained on the stabilized compound set using 3 tasks. In the first task, which involves atom prediction, 15% of the input atoms were randomly masked. The model then predicted the types of masked atoms based on the information from the surrounding atoms and their 3D coordinates. The loss for the atom prediction task, $l_{\mathrm{atom}\;}$, is expressed in Equation (1):


(1) latom =-1|nmask|∑i∈nmasklog p(yi|hi),

where $n_{\mathrm{mask}}$ denotes the set of indices of masked atoms, $y_{i}$ denotes the ground-truth atom type label of atom i, $h_{i}$ denotes the embedding representation of atom i produced by the model, and $p(y_{i}|h_{i})\;$represents the probability that atom i is of type $y_{i}\;$given its embedding representation ${\;h}_{i}$.

In the second task, which involves coordinate reconstruction, independent noise of ±1 Å was added to each coordinate dimension of each atom in the input 3D structures. The model was trained to estimate the noise components and reconstruct the original coordinates. The prediction loss for this task, $l_{\mathrm{coord}}$, is defined as the Smooth L1 (SL1) loss over all atoms, as expressed in Equation (2).


(2) $$l_{\mathrm{coord}}=\sum_{i=1}^{n}\mathrm{SL}1(c_{i},{\hat{c}}_{i})\;=\sum_{i=1}^{n}\left(\mathrm{SL}1(x_{1i},{\hat{x}}_{1i})+\mathrm{SL}1(x_{2i},{\hat{x}}_{2i}\right)+SL1(x_{3i},{\hat{x}}_{3i})),$$

where n denotes the number of atoms in a compound, and $c_{i}=(x_{1i},\;x_{2i},\;x_{3i}{)}^{T}\;$and ${\hat{\;c}}_{i}=({\hat{x}}_{1i},\;{\hat{x}}_{2i},\;{\hat{x}}_{3i}{)}^{T}\;$denote the ground truth and predicted 3D coordinates of atom *i*, respectively. The SL1 loss in this task can be expressed in Equation (3):


(3) SL1(x1i, x^1i)={  12(x1i- x^1i)2if  |x1i- x^1i| ≤1|x1i- x^1i|- 12otherwise

In the third task, which involves atom-pair distance prediction, atom-pair distances computed from the noise-added 3D coordinates were used as input. The model was trained to reconstruct the original atom-pair distances. The prediction loss for this task, $l_{\mathrm{dist}}$, is defined as the SL1 loss over all atom pairs, as expressed in Equation (4):


(4) ldist=∑i=1n∑j=i+1nSL1(ri,j,r^i,j),

where$\;r_{i,j}\;$and$\;{\hat{r}}_{i,j}\;$denote the ground truth and predicted Euclidean distances between atoms i and j, respectively. The SL1 function in this task can be expressed in Equation (5):


(5) SL1(ri,j, r^i,j)={12(ri,j- r^i,j)2 if  |ri,j- r^i,j| ≤1|ri,j- r^i,j|- 12otherwise

The model parameters were updated to minimize the sum of the losses from the 3 tasks. For each compound, a structurally stabilized 3D conformation was obtained using a Uni-Mol model fine-tuned on the GEOM-Drugs dataset (Axelrod and Gómez-Bombarelli 2022). The stabilized 3D conformation was then fed into the pretrained Uni-Mol model, and a 512-dimensional embedding representation, denoted as u, was extracted.

### 2.5 Construction of compound graphs on 3D coordinates

We constructed compound graphs based on 3D structures to capture spatial relationships among atoms. Specifically, we defined a compound graph G=(V, E) based on the 3D atomic coordinates generated by the Uni-Mol model fine-tuned on the GEOM-Drugs dataset (Axelrod and Gómez-Bombarelli 2022). The node set V corresponds to atoms in a compound. To construct the edge set E, pairwise Euclidean distances were computed from atomic coordinates, and atom pairs whose distances were within 6.0 Å were connected by edges. This distance threshold was selected to capture both covalent and non-covalent interactions between atoms (Shen *et al.* 2023).

For node feature construction, the CanonicalAtomFeaturizer provided by DGL-LifeSci (Li, *et al.* 2021) was used. For each atom i, a 74-dimensional feature vector was generated by encoding the atom type, hybridization state (SP, SP2, SP3, SP3D, and SP3D2), formal charge, aromaticity, and number of hydrogen atoms. The dimensionality of this feature vector was large relative to the number of encoded atomic properties because most of these properties were represented using one-hot encoding.

### 2.6 Embedding representations on 3D compound graphs

We obtained compound embedding representations based on 3D compound graphs using GAT. First, each node feature vector was mapped to a 128-dimensional vector via linear transformation. Next, 3 GAT layers were applied. On each layer, the node feature vector was updated based on the neighborhood set of nodes weighted by attention coefficients. The attention coefficient was computed by applying a trainable attention vector to the concatenated feature vector of a pair of nodes. In the intermediate layers, we used multi-head attention with 4 heads and concatenated the outputs of all heads. As the output, we obtained an embedding representation $Q\;\in \;R^{\left(n\times 128\right)}$, where n denotes the number of atoms.

### 2.7 Integration of compound embedding representations

We integrated 2 compound embeddings to obtain a unified, structure-informed representation. We integrated 2 compound embeddings: Q and u. Q was obtained from the 3D compound graph, and u was obtained from Uni-Mol pretrained on the structurally stabilized compound set. First, u was transformed using 2 fully connected layers. Next, L2 normalization was applied, and $u\prime \in \;R^{128}$ was obtained. Finally, the scaled u′ was added to the original graph-based embedding Q. This process can be expressed in Equation (6):


(6) M=(Qu')u'T+Q,

where $M\;\in \;R^{\left(m\times 128\right)}\;$denotes the compound feature vector obtained by integrating the embedding representation based on the 3D compound graph with the embedding representation obtained from Uni-Mol pretrained on structurally stabilized compounds.

### 2.8 Construction of protein feature vectors with 3D structural information

We constructed protein feature vectors that incorporate 3D structural information using multiple protein language models. First, an amino acid sequence was input into ProstT5, a protein language model, to obtain a 3Di sequence, which is a string representation containing 3D structural information. Next, the generated 3Di sequence and the amino acid sequence were alternately arranged to obtain a structure-aware sequence. The structure-aware sequence was then input into SaProt, a structure-aware protein language model, and an embedding representation of the protein was extracted. The dimension of the embedding representation produced by SaProt was then reduced using 2 fully connected layers to construct the final protein feature vector $e\;\in \;R^{128}$.

### 2.9 Fusion of compound and protein representations

After constructing compound and protein feature vectors, they were fused using BAN. First, the compound feature $M\;\in \;R^{\left(n\times 128\right)}$ and protein feature $e\;\in \;R^{128}$ were first transformed by linear layers to obtain $M^{\prime }\in \;R^{\left(n\times 768\right)}$ and ${\;e}^{\prime }\in \;R^{768}$, respectively. Second, using $M^{\prime }$ and ${\;e}^{\prime }$, a bilinear interaction map was designed. Third, a bilinear pooling layer produces an integrated compound–protein representation $b\;\in \;R^{768}$ from the bilinear interaction map. Finally, the dimension of the integrated representation, b, was further reduced via sum pooling with stride 3, obtaining compound–protein fused feature vector $b^{\prime }\in \;R^{256}$. We primarily determined the dimensionality of the embeddings with reference to the GraphBAN architecture (Hadipour *et al.* 2025).

### 2.10 Domain adaptation for unseen compounds and proteins

To improve generalization performance for unseen compounds and proteins excluded from the training data, we introduced CDAN following a previous study (Hadipour *et al.* 2025). The CDAN framework consists of 3 neural networks: a feature extractor including the BAN layer, an interaction predictor, and a domain discriminator. The feature extractor generates a fused feature vector b′ for each compound–protein pair, and the interaction predictor applies a softmax function to b′ and outputs an interaction probability $p\;\in \;R^{2}$. The domain discriminator receives a conditional representation z, which is obtained by combining b′ and p, and classifies whether the input comes from the source or target domain.

During training, a cross-entropy loss $l_{\mathrm{cls}}$ was computed using labeled samples from the source domain. This loss was computed from the predicted interaction probabilities and ground-truth labels. Next, conditional representations $z_{s}$ and $z_{t}$ were constructed for the source and target domains, respectively. Using these representations, we computed the adversarial loss $l_{\mathrm{adv}}$ expressed in Equation (7):


(7) ladv(B,P,D)=Ext log (1−D(zt))+Exs log (1−D(zs)),

where *B* is a feature extractor that generates the fused feature vector ***b′*** for each compound–protein pair, *P* is an interaction predictor that outputs an interaction probability vector p from b′, and *D* is a domain discriminator that takes the conditional representation ***z*** as input and specifies whether the input comes from the source or target domains. The terms $E_{x^{s}}$ and $E_{x^{t}}$ denote the average over mini batches sampled from the source and target domains, respectively.

This adversarial learning can be formulated as the min–max optimization problem (Equation (8)). In this training, the CPI prediction loss on the source domain, ${\;l}_{\mathrm{cls}}$, is minimized, while domain-dependent information is suppressed.


(8) lCDAN=minD⁡maxF,G⁡lcls(F,G)-β ladv(F,G,D),

where β is a hyperparameter that controls the balance between task learning and domain adaptation. In our experiments, we set *β* to 0 for the first 9 epochs and to 1 from epoch 10 onward, over a total of 50 training epochs.

### 2.11 Interaction prediction using the CPI network structure

We adopted the KD framework used in GraphBAN to leverage CPI network information in the training data. This framework comprises 2 stages: pretraining the teacher model and training the student model.

The teacher model represents the CPIs in the training data as a network. Compounds and proteins are treated as nodes, and interactions are treated as edges, forming a bipartite graph. A GAE was employed to learn the network structure. The initial representations of compound and protein nodes were given as 128-dimensional one-hot vectors. The encoder consists of 3 GraphSAGE layers (Hamilton *et al.* 2017), which aggregate information from neighboring nodes to capture the network structure.

The student model performs interaction prediction based on 3D structural features extracted from compounds and proteins while incorporating the network knowledge learned by the teacher model. The KD loss $l_{\mathrm{KD}}$ is expressed in Equation (9):


(9) lKD=1nbatch∑i=1nbatch‖b′i- ti‖2,

where $n_{\mathrm{batch}}$ is the batch size.

The overall loss of the student model, $l_{\mathrm{total}}$, is defined as the sum of the CPI prediction loss $l_{\mathrm{cls}}$ and the KD loss $l_{\mathrm{KD}}$, to minimize $l_{\mathrm{cls}}+l_{\mathrm{KD}}$.

Details about the hyperparameters and experimental settings are provided in the “Experimental Settings” section of the Supplementary Methods, available as supplementary data at *Bioinformatics* online.

## 3 Results

### 3.1 Performance of CPI prediction using 3D structural information under the inductive scenario

We evaluated the performance of CPI prediction. We compared DESTIN with existing methods: Random Forest (Breiman, 2001), MolTrans (Huang *et al.* 2021), DeepConv-DTI (Lee *et al.* 2019), SPVec-SGN-CPI (Zhang *et al.* 2024), GraphDTA (Nguyen *et al.* 2021), GraphsformerCPI (Ma *et al.* 2024), DrugBAN (Bai *et al.* 2023), DSANIB (Tian *et al.* 2025), PocketDTA (Zhao *et al.* 2024), FusionDTI (Meng *et al.* 2025), and GraphBAN (Hadipour *et al.* 2025) under the inductive scenario. The results of these existing methods were directly obtained from the original GraphBAN paper (Hadipour *et al.* 2025).

Table 2A lists the results for BindingDB. DESTIN exhibited the highest AUC and F1 score. However, GraphBAN recorded the highest AUPR, followed by DESTIN. Tables 2B and 2C present the results for BioSNAP and PDBbind 2016, respectively. DESTIN exhibited the best performance on all metrics. Table 2D shows the results for KIBA. DESTIN did not exhibit the best performance on this dataset. GraphBAN, GraphsformerCPI, and DrugBAN exhibited the highest AUC, AUPR, and F1 score, respectively. These results indicate that 3D structural information improves prediction performance even for unseen compounds and proteins; however, the benefit may be limited for datasets biased toward specific protein families, such as KIBA. The usefulness of DESTIN was also observed under the transductive scenario (Table S2, available as supplementary data at *Bioinformatics* online).

**Table 2:** Performance evaluation of CPI prediction methods under the inductive scenario for different datasets: (A) BindingDB, (B) BioSNAP, (C) PDBbind 2016, and (D) KIBA.

Model	AUC	AUPR	F1
**(A) Performance evaluation on the BindingDB dataset**			

RF	0.523 ± 0.061	0.428 ± 0.037	0.667 ± 0.005
MolTrans	0.518 ± 0.048	0.436 ± 0.057	0.667 ± 0.001
DeepConv-DTI	0.547 ± 0.021	0.445 ± 0.054	0.525 ± 0.017
SPVec-SGCN-CPI	0.558 ± 0.016	0.449 ± 0.018	0.670 ± 0.010
GraphDTA	0.541 ± 0.025	0.453 ± 0.031	0.668 ± 0.000
GraphsformerCPI	0.552 ± 0.008	0.460 ± 0.044	0.522 ± 0.024
DrugBAN	0.586 ± 0.043	0.489 ± 0.073	0.672 ± 0.007
DSANIB	0.506 ± 0.032	0.426 ± 0.052	0.667 ± 0.001
PocketDTA	0.554 ± 0.012	0.450 ± 0.011	0.669 ± 0.009
FusionDTI	0.514 ± 0.043	0.425 ± 0.021	0.432 ± 0.070
GraphBAN	0.618 ± 0.036	**0.532 ± 0.056**	0.688 ± 0.018
DESTIN	**0.627 ± 0.033**	0.515 ± 0.060	**0.694 ± 0.016**

**(B) Performance evaluation on the BioSNAP dataset**			

RF	0.624 ± 0.032	0.613 ± 0.035	0.673 ± 0.006
MolTrans	0.655 ± 0.016	0.671 ± 0.019	0.672 ± 0.007
DeepConv-DTI	0.624 ± 0.032	0.613 ± 0.035	0.673 ± 0.006
SPVec-SGCN-CPI	0.626 ± 0.012	0.617 ± 0.017	0.673 ± 0.009
GraphDTA	0.694 ± 0.012	0.728 ± 0.008	0.678 ± 0.007
GraphsformerCPI	0.620 ± 0.026	0.598 ± 0.026	0.681 ± 0.009
DrugBAN	0.687 ± 0.029	0.748 ± 0.032	0.672 ± 0.006
DSANIB	0.554 ± 0.016	0.551 ± 0.018	0.669 ± 0.002
PocketDTA	0.629 ± 0.011	0.620 ± 0.011	0.666 ± 0.014
FusionDTI	0.672 ± 0.021	0.687 ± 0.021	0.501 ± 0.105
GraphBAN	0.751 ± 0.023	0.807 ± 0.018	0.697 ± 0.017
DESTIN	**0.806 ± 0.008**	**0.821 ± 0.014**	**0.743 ± 0.011**

**(C) Performance evaluation on the PDBbind 2016 dataset**			

RF	0.533 ± 0.062	0.560 ± 0.042	0.666 ± 0.005
MolTrans	0.542 ± 0.041	0.559 ± 0.029	0.671 ± 0.003
DeepConv-DTI	0.543 ± 0.052	0.544 ± 0.042	0.489 ± 0.038
SPVec-SGCN-CPI	0.551 ± 0.048	0.544 ± 0.040	0.657 ± 0.027
GraphDTA	0.567 ± 0.030	0.556 ± 0.038	0.673 ± 0.003
GraphsformerCPI	0.523 ± 0.069	0.531 ± 0.063	0.509 ± 0.075
DrugBAN	0.557 ± 0.043	0.566 ± 0.024	0.670 ± 0.004
DSANIB	0.518 ± 0.042	0.532 ± 0.017	0.672 ± 0.005
PocketDTA	0.557 ± 0.020	0.545 ± 0.018	0.667 ± 0.020
FusionDTI	0.598 ± 0.062	0.597 ± 0.026	0.474 ± 0.074
GraphBAN	0.561 ± 0.055	0.560 ± 0.042	0.677 ± 0.010
DESTIN	**0.721 ± 0.040**	**0.708 ± 0.059**	**0.727 ± 0.018**

**(D) Performance evaluation on the KIBA dataset**			

RF	0.626 ± 0.015	0.285 ± 0.051	0.667 ± 0.003
MolTrans	0.598 ± 0.023	0.246 ± 0.028	0.676 ± 0.004
DeepConv-DTI	0.622 ± 0.017	0.305 ± 0.018	0.490 ± 0.045
SPVec-SGCN-CPI	0.627 ± 0.007	0.299 ± 0.008	0.648 ± 0.009
GraphDTA	0.631 ± 0.042	0.314 ± 0.070	0.672 ± 0.003
GraphsformerCPI	0.633 ± 0.006	**0.379 ± 0.014**	0.517 ± 0.067
DrugBAN	0.633 ± 0.019	0.275 ± 0.055	**0.678 ± 0.006**
DSANIB	0.601 ± 0.033	0.256 ± 0.057	0.669 ± 0.001
PocketDTA	0.629 ± 0.008	0.291 ± 0.010	0.648 ± 0.012
FusionDTI	0.582 ± 0.008	0.249 ± 0.030	0.206 ± 0.061
GraphBAN	**0.654 ± 0.017**	0.306 ± 0.041	0.677 ± 0.005
DESTIN	0.623 ± 0.014	0.274 ± 0.032	0.672 ± 0.003

Values are reported as mean ± SD over 5 runs using different random seeds (12, 14, 16, 18, and 20). The highest value for each metric is shown in bold.

### 3.2 Identification of protein categories with improved prediction performance using 3D structural information

To investigate the characteristics of DESTIN’s predictions, we compared the prediction performance of DESTIN and GraphBAN at the protein category level. For each protein category, we counted the number of proteins for which DESTIN or GraphBAN outperformed the other method. Details of the protein category annotation procedure are provided in the “Protein category annotation using the UniProt REST API” section of the Supplementary Methods, available as supplementary data at *Bioinformatics* online.

Figure 3 shows the number of proteins for which each method exhibited superior performance in each protein category, based on the BindingDB dataset. Across different categories, including enzyme, protein kinase, and ion channel, DESTIN exhibited higher prediction accuracy than GraphBAN for a greater number of proteins. Notably, DESTIN outperformed GraphBAN for the major membrane protein categories such as ion channels and transporters.

**Figure 3:**
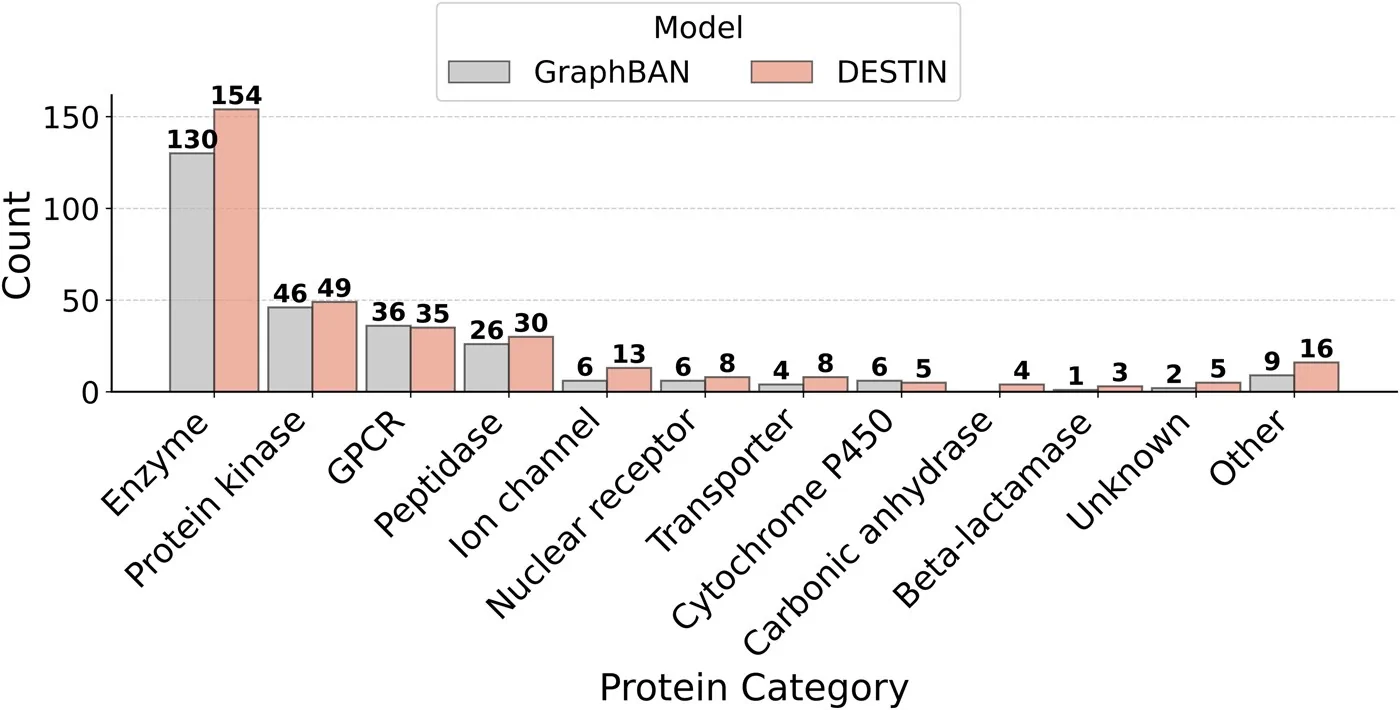
Protein category distributions based on model-wise differences in prediction performance for BindingDB. The gray bars indicate the number of proteins for which GraphBAN exhibited higher AUC values, and the red bars indicate the number of proteins for which DESTIN exhibited higher AUC values. Proteins with protein-level AUC ≥ 0.5 were used for this analysis, as AUC < 0.5 indicates performance no better than random prediction. Proteins were grouped by UniProt category based on the method that exhibited higher AUC values.

Similar trends were observed for the BioSNAP dataset, where DESTIN outperformed GraphBAN for the major membrane protein categories such as GPCRs and ion channels (Fig. S2, available as supplementary data at *Bioinformatics* online). In contrast, GraphBAN outperformed DESTIN across all categories in the KIBA dataset (Fig. S3, available as supplementary data at *Bioinformatics* online).

Membrane proteins constitute more than 50% of all therapeutic targets and represent an extremely important category in drug discovery (Overington *et al.* 2006; Santos *et al.* 2017). They comprise major drug target classes, such as GPCR and ion channels. However, membrane proteins have complex 3D structures embedded within lipid bilayers, making crystallization difficult and resulting in limited experimentally determined structural information. Consequently, conventional CPI prediction methods based solely on amino acid sequence information cannot adequately capture structural features, particularly those associated with transmembrane regions and ligand-binding sites. DESTIN exhibited significantly improved predictive performance for membrane proteins across the BindingDB and BioSNAP datasets, indicating that the complex structural characteristics unique to membrane proteins can be more effectively captured by incorporating 3D structural information in the model.

Overall, these results show that the proposed model is a promising approach for drug discovery targeting membrane proteins, which are difficult to accurately predict using conventional methods due to their structural complexity.

### 3.3 Examples of proteins showing marked differences in predictive performance between models

We analyzed proteins that exhibited large differences in AUC values between DESTIN and GraphBAN. Figure 4 shows the proteins for which DESTIN substantially outperformed GraphBAN, including the vasopressin V1a receptor (AUC difference: 0.455), cysteinyl leukotriene receptor 1 (difference: 0.377), and glutamate receptor ionotropic NMDA 2B (difference: 0.428). For these proteins, DESTIN exhibited high predictive performance with AUC values exceeding 0.9, whereas GraphBAN exhibited AUC values of approximately 0.5, resulting in AUC differences of 0.4–0.5.

**Figure 4:**
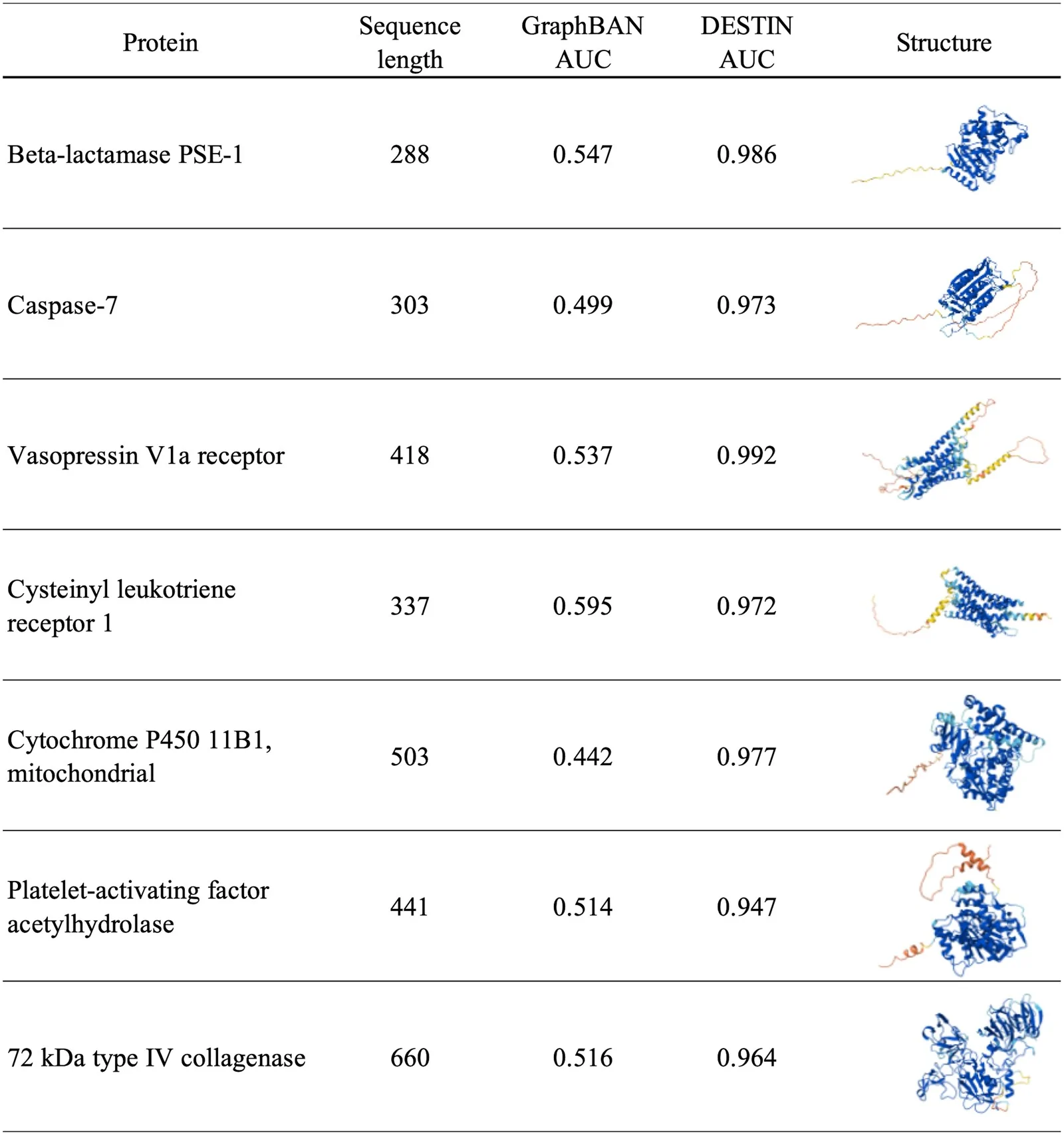
Examples of proteins with pronounced differences in predictive performance between DESTIN and GraphBAN. Proteins for which DESTIN outperformed GraphBAN are shown. In the structures, orange regions indicate residues with low-confidence predictions by AlphaFold2.

The proteins for which DESTIN outperformed GraphBAN include membrane proteins, such as GPCRs and ligand-gated ion channels. For example, cysteinyl leukotriene receptor 1, a membrane protein, is widely known as a therapeutic target of the asthma drug, montelukast (Lynch *et al*. 1999). These results indicate that the high predictive performance of DESTIN for structurally complex membrane proteins can be attributed to the incorporation of 3D structural information, which is effective for capturing drug-binding modes of proteins with complex transmembrane architectures.

## 4 Discussion

In this study, we developed DESTIN, a CPI prediction framework based on the 3D structures of compounds and proteins. The proposed framework uses 3D information on both sides and does not require extensive coordinate preparation or computationally expensive simulations. The proposed framework enables stable prediction performance for new compounds and proteins absent in the training set. It also improves accuracy even for structurally complex and pharmacologically important membrane proteins. DESTIN can be employed in various pharmaceutical applications, such as hit discovery and off-target screening.

Membrane proteins with complex 3D structures were further examined. Membrane proteins are embedded in lipid bilayers; thus, only sequence information is often insufficient because it may not well represent transmembrane regions or miss ligand-binding environments. DESTIN performed well on membrane proteins, indicating that the model can effectively capture the 3D structural features even for complex proteins.

## 5 Limitations

This study has several limitations. First, accurately representing compound structures remains challenging. In this study, compound structures were generated by stabilizing RDKit conformers. Because these conformers do not account for solvent effects or binding-induced conformational changes, predictive accuracy may decrease for highly flexible ligands and protein-ligand systems exhibiting substantial induced fit. Second, model performance may be limited for proteins that are not accurately represented by the ProstT5-based 3Di representation, as the discretization process can result in information loss. Therefore, developing more informative structural representations for both compounds and proteins remains an important direction for future research.

Furthermore, prediction accuracy may be reduced for proteins whose structures are poorly predicted by AlphaFold, because DESTIN relies on foundation models trained on protein representations derived from AlphaFold-predicted structures. In addition, as several previous methods outperformed DESTIN on the KIBA dataset, the proposed approach may be less suitable for kinase-focused prediction tasks.

Finally, CPI datasets such as BindingDB have inherent limitations regarding the conversion and integration of experimental values obtained under different assay conditions. Such heterogeneity may introduce inconsistencies in the reported binding data. Although we used the same binarized datasets as those employed by GraphBAN (Hadipour *et al.* 2025) to ensure a fair comparison, a detailed investigation of the model’s ability to discriminate among different types and magnitudes of experimental measurements would be another direction for future work.

## Supplementary Material

btag710_Supplementary_Data

## Data Availability

The data supporting this study are publicly available on GitHub and archived on Zenodo (https://doi.org/10.5281/zenodo.21759701).
