# Supplementary material for: Genome-wide-scale prediction of compound−protein interactions using foundation and language models based on three-dimensional structures of compounds and proteins
Source: Bioinformatics. 2026 Sep 28;42(10):btag710. doi: 10.1093/bioinformatics/btag710 (PMC13674696; doi:10.1093/bioinformatics/btag710)
Supplement: Supplementary file 1 [file btag710_supplementary_data.pdf]

Supplementary information for

Genome-wide-scale prediction of compound–protein interactions using foundation and language models based on three-dimensional structures of compounds and proteins

Yuga Moriyama[1,+], Yuki Matsukiyo[1,+], Tsuyoshi Kimura[1], Hiyori Yamamoto[1], Yuko Sakajiri[1], Tomokazu Shibata[1], Ryusuke Sawada[2] and Yoshihiro Yamanishi[1,3,*]

[1]Department of Complex Systems Science, Graduate School of Informatics, Nagoya University, Nagoya, Aichi 464-8601, Japan.

[2]Integrated Glyco-Big Data Center (iGDATA), Institute for Glyco-core Research (iGCORE), Nagoya University, Nagoya, Aichi 464-8601, Japan

[3]Division of Interdisciplinary Research and Development (R&D), Aichi Cancer Center Research Institute, Nagoya, Aichi 464-8681, Japan.

Supplementary Methods

Experimental settings

DESTIN was implemented using Python 3.9.13 and PyTorch 2.3.1. Compound graph processing was performed using Deep Graph Library version 2.4.0. The graph autoencoder (GAE) used for KD was implemented with PyTorch Geometric version 2.6.1. Compound structure preprocessing was conducted using RDKit version 2023.3.2. For protein SA embeddings, Rostlab/ProstT5_fp16 was used to infer 3Di sequences, and westlake-repl/SaProt_1.3B_AFDB_OMG_NCBI was used to generate embeddings. All experiments were conducted on an NVIDIA Tesla T4 GPU using the settings and hyperparameters listed in Supplementary Table 1. The computational time required for each step of the proposed workflow are also summarized in Supplementary Table 2.

Supplementary Table 1: Hyperparameters and experimental settings for DESTIN training. The table summarizes the hyperparameters for each major model component, including the protein encoder, compound encoders (GAT-based and pretrained Uni-Mol embeddings), bilinear attention network (BAN), graph autoencoder (GAE), and the teacher and student modules used for knowledge distillation and domain adaptation.

Module	Hyperparameter	Value
Full model	Batch size	32
Protein encoder	SaProt embedding dimension	1280
	MLP hidden dimension	512
	Output dimension	128
	Dropout rate	0.1
Molecule encoder (GAT)	GAT layers	3
	Heads of attention	4
	Initial atom embedding dimension	128
	Output dimension	128
Molecule encoder	Uni-Mol embedding dimension	512
(Pretrained Uni-Mol embedding)	Linear layers	2
	Hidden dimension	512
	Output dimension	128
	Dropout rate	0.5
BAN	Heads of bilinear attention	2
MLP decoder	Input dimension	256
	Hidden dimension	512
	Output dimension	128
GAE	GraphSAGE layers	3
	Hidden dimension	128
	Dropout rate	0.5
	Linear layers	1
Teacher module	Learning rate	1e-3
	Max epochs	500
Student module	Learning rate	1e-4
	Maximum epochs	50
Student module - CDAN	Learning rate	5e-5
	Init epoch	10

Supplementary Table 2: Computational time required for each step of the proposed workflow. All times are reported in hh:mm:ss format, where hh, mm, and ss denote hours, minutes, and seconds, respectively. For compound feature extraction and protein 3Di sequence generation, the reported values represent the time required to process all unique compounds and proteins in each dataset. For teacher and student model training, the computational times were measured using the inductive scenario dataset (seed = 12).

Step	BindingDB	BioSNAP	PDBbind2016	KIBA
Compound feature extraction	26:41:23	19:21:41	4:25:47	1:33:08
Protein 3Di sequence generation	18:13:10	13:41:03	8:23:03	1:57:05
Teacher model training	0:00:36	0:00:14	0:00:16	0:00:19
Student model training	3:20:56	1:55:44	0:40:41	7:18:34
Total	48:16:05	34:58:42	13:29:47	10:49:06

Dataset

BindingDB (Gilson et al., 2016) is a public database of experimentally measured binding affinities collected from various sources, such as scientific articles and US patents. In this study, we used a CPI dataset constructed by Huang et al. (2021). Approximately 50,000 CPI entries were extracted from this dataset following the same procedure de-scribed by Hadipour et al. (2025). BioSNAP (Zitnik et al., 2018) is a drug–target interaction network dataset extracted from DrugBank (Wishart et al., 2008). PDBbind 2016 (Cang et al., 2018) is a dataset of protein–ligand complex structures registered in the Protein Data Bank (PDB), with experimentally measured binding affinities assigned to each complex. KIBA (Tang et al., 2014) is a kinase inhibitor dataset in which multiple bioassay measurements (e.g., IC50, Ki, and Kd) are converted into a unified score.

Dataset splitting for performance evaluation

In the inductive scenario (Fig. 2(A)), we evaluated the generalization performance for unseen compounds and proteins. Following GraphBAN (Hadipour et al., 2025), we performed clustering-based domain splitting. Extended connectivity fingerprints (ECFP) (Rogers and Hahn, 2010) and Jaccard distance were used for compounds, and k-mer (k = 3) frequency vectors and cosine distance were used for proteins. Hierarchical clustering was performed using the single-linkage method for both compounds and proteins. Next, 40% of the clusters were selected as the target domain, and the remaining 60% were used as the source domain. The source domain contains only compound–protein pairs in which both the compound and protein clusters belong to the source domain. Similarly, the target domain contains only pairs in which both belong to the target domain. The samples in the target domain were then split into training (80%) and test (20%) sets. Labeled data from the source domain were used to train the interaction prediction task as a binary classification problem. The training data in the target domain were used for domain adaptation using CDAN.

Details about construction of compound graphs on 3D coordinates

We constructed compound graphs based on 3D structures to capture spatial relationships among atoms. Specifically, we defined a compound graph $G = (V, E)$ based on the 3D atomic coordinates generated by the Uni-Mol model fine-tuned on the GEOM-Drugs dataset (Axelrod et al., 2022). The node set V corresponds to atoms in a compound. To construct the edge set E, pairwise Euclidean distances were computed from atomic coordinates, and atom pairs whose distances were within 6.0 Å were connected by edges. This distance threshold was selected to capture both covalent and non-covalent interactions between atoms.

This distance threshold was selected to capture both covalent and non-covalent interactions between atoms. Covalent bonds are typically formed at distances of approximately 0-2 Å, whereas hydrogen bonds and van der Waals interactions are generally observed at distances of approximately 2-4 Å and 4-6 Å, respectively (Shen et al., 2023). Therefore, a cutoff distance of 6 Å was used to encompass these chemically relevant interactions within compounds.

Following a previous study (Hadipour et al., 2025), we used the CanonicalAtomFeaturizer provided by DGL-LifeSci (Li, M. et al., 2021) to construct node feature vectors. For each atom i, a 74-dimensional feature vector was generated by encoding the atom type, hybridization state (SP, SP2, SP3, SP3D, and SP3D2), formal charge, aromaticity, and number of hydrogen atoms. The dimensionality of the resulting feature vector was large relative to the number of atomic properties encoded because most of these properties were represented using one-hot encoding. For example, the atom type alone was represented as a 43-dimensional one-hot vector.

Protein category annotation using the UniProt REST API

For protein category annotation, we used similarity comments and EC numbers obtained by submitting amino acid sequences to the UniProt REST API and identifying entries with identical sequences and lengths. Similarity comments describe protein family membership (e.g., "Belongs to the calcium channel alpha-1 subunit family"). Accordingly, protein categories were assigned on the basis of these descriptions using predefined keyword-based criteria. For example, ion channels were assigned based on the presence of terms such as “ion channel”, “sodium channel”, “calcium channel”, or “P2X receptor” in the similarity comments. The correspondence between protein categories and the keywords used for their annotation is summarized in Supplementary Table 3.

Proteins with an EC number were additionally assigned to the Enzyme category. The Other category included proteins with similarity comments that did not match any predefined category and were not assigned to the Enzyme category based on an EC number. The Unknown category included proteins for which neither similarity comments nor EC numbers were available.

Supplementary Table 3: Correspondence between protein categories and keywords used for annotation. For each protein category, proteins were assigned when the similarity comment retrieved from UniProt contained any of the listed keywords, except for the Nuclear receptor category, which required the presence of both “nuclear” and “receptor”.

Protein category	Keywords
GPCR	g-protein coupled
Ion channel	ion channel, ionotropic, sodium channel, calcium channel, potassium channel, chloride channel, p2x receptor, transient receptor
Transporter	transporter, symporter, antiporter, solute carrier
Protein kinase	kinase
Peptidase	peptidase, protease
Cytochrome P450	cytochrome p450
Carbonic anhydrase	carbonic anhydrase
Beta-lactamase	beta-lactamase
Nuclear receptor	nuclear, receptor

Supplementary Results

Performance of CPI prediction using 3D structural information under the transductive scenario

We evaluated the performance of all methods under the transductive scenario, and the results are listed in Supplementary Table 4.

Supplementary Table 4(A) lists the results for the BindingDB dataset. Random Forest (RF) (Breiman, 2001) exhibited the highest AUC (0.973 ± 0.002) and AUPR (0.966 ± 0.003), and SPVec-SGCN-CPI (Wang et al., 2022) recorded the highest F1 score (0.941 ± 0.019).

Supplementary Table 4(B) lists the results for the BioSNAP dataset. DESTIN exhibited the best performance across all evaluation metrics, with an AUC value of 0.935 ± 0.005, an AUPR value of 0.932 ± 0.011, and an F1 score of 0.876 ± 0.004. Compared with DrugBAN (AUC 0.924 ± 0.007), DESTIN improved the AUC value by 0.011.

Supplementary Table 4(C) lists the results for the PDBbind 2016 dataset. DESTIN exhibited the highest AUC (0.892 ± 0.012) and F1 score (0.832 ± 0.009), but RF recorded the highest AUPR (0.847 ± 0.008).

Supplementary Table 4(D) lists the results for the KIBA dataset. DrugBAN exhibited the highest AUC (0.937 ± 0.008) and F1 score (0.860 ± 0.003), and DESTIN recorded the second-best AUC (0.927 ± 0.005).

Overall, DESTIN exhibited the best performance on all metrics for BioSNAP and showed the best AUC and F1 score for PDBbind 2016. Although DESTIN did not exhibit the highest performance on BindingDB and KIBA, the performance gaps were limited. These results indicate that incorporating 3D structural information effectively improves the performance of prediction models under the transductive scenario.

Supplementary Table 4: Performance evaluation of CPI prediction methods that utilize 3D structural information under the transductive scenario for datasets: (A) BindingDB, (B) BioSNAP, (C) PDBbind 2016, and (D) KIBA. Values are reported as mean ± standard deviation over five runs using different random seeds (12, 14, 16, 18, and 20), and the best value for each metric is shown in bold. For GraphBAN, the inductive architecture was adopted. For FusionDTI, some interaction entries are missing. The missing rates are 6.0% for BioSNAP, 4.2% for BindingDB, 4.0% for KIBA, 9.4% for C. elegans, and 6.1% for PDBbind 2016.

(A) Performance evaluation on the BindingDB dataset

Model	AUC	AUPR	F1
RF	**0.973 ± 0.002**	**0.966 ± 0.003**	0.914 ± 0.003
MolTrans	0.961 ± 0.003	0.950 ± 0.005	0.910 ± 0.003
DeepConv-DTI	0.938 ± 0.005	0.915 ± 0.007	0.872 ± 0.004
SPVec-SGCN-CPI	0.971 ± 0.011	0.965 ± 0.011	**0.941 ± 0.019**
GraphDTA	0.969 ± 0.006	0.948 ± 0.010	0.923 ± 0.016
GraphformerCPI	0.960 ± 0.004	0.953 ± 0.004	0.906 ± 0.004
DrugBAN	0.966 ± 0.003	0.955 ± 0.003	0.916 ± 0.003
DSANIB	0.959 ± 0.005	0.952 ± 0.003	0.910 ± 0.005
PocketDTA	0.934 ± 0.020	0.907 ± 0.033	0.833 ± 0.061
FusionDTI	0.962 ± 0.003	0.949 ± 0.003	0.907 ± 0.004
GraphBAN	0.959 ± 0.005	0.946 ± 0.007	0.898 ± 0.005
DESTIN	0.961 ± 0.005	0.945 ± 0.008	0.904 ± 0.004

(B) Performance evaluation on the BioSNAP dataset

Model	AUC	AUPR	F1
RF	0.880 ± 0.008	0.899 ± 0.009	0.814 ± 0.005
MolTrans	0.898 ± 0.008	0.905 ± 0.011	0.832 ± 0.010
DeepConv-DTI	0.891 ± 0.007	0.898 ± 0.006	0.821 ± 0.018
SPVec-SGCN-CPI	0.873 ± 0.027	0.878 ± 0.034	0.810 ± 0.021
GraphDTA	0.885 ± 0.014	0.868 ± 0.005	0.819 ± 0.013
GraphformerCPI	0.872 ± 0.007	0.886 ± 0.008	0.813 ± 0.004
DrugBAN	0.924 ± 0.007	0.930 ± 0.012	0.855 ± 0.006
DSANIB	0.865 ± 0.010	0.874 ± 0.009	0.810 ± 0.004
PocketDTA	0.922 ± 0.013	0.922 ± 0.010	0.847 ± 0.011
FusionDTI	0.903 ± 0.003	0.909 ± 0.007	0.834 ± 0.001
GraphBAN	0.905 ± 0.008	0.912 ± 0.010	0.836 ± 0.006
DESTIN	**0.935 ± 0.005**	**0.932 ± 0.011**	**0.876 ± 0.004**

(C) Performance evaluation on the PDBbind 2016 dataset

Model	AUC	AUPR	F1
RF	0.809 ± 0.015	**0.847 ± 0.008**	0.730 ± 0.018
MolTrans	0.764 ± 0.007	0.769 ± 0.018	0.721 ± 0.007
DeepConv-DTI	0.644 ± 0.018	0.652 ± 0.020	0.680 ± 0.008
SPVec-SGCN-CPI	0.815 ± 0.026	0.840 ± 0.013	0.721 ± 0.015
GraphDTA	0.817 ± 0.025	0.844 ± 0.016	0.739 ± 0.016
GraphformerCPI	0.802 ± 0.007	0.837 ± 0.007	0.728 ± 0.015
DrugBAN	0.820 ± 0.012	0.829 ± 0.006	0.753 ± 0.011
DSANIB	0.805 ± 0.007	0.835 ± 0.009	0.723 ± 0.007
PocketDTA	0.827 ± 0.027	0.803 ± 0.023	0.752 ± 0.030
FusionDTI	0.748 ± 0.015	0.754 ± 0.024	0.707 ± 0.008
GraphBAN	0.752 ± 0.024	0.773 ± 0.017	0.712 ± 0.016
DESTIN	**0.892 ± 0.012**	0.884 ± 0.018	**0.832 ± 0.009**

(D) Performance evaluation on the KIBA dataset

Model	AUC	AUPR	F1
RF	0.915 ± 0.003	0.762 ± 0.009	0.839 ± 0.005
MolTrans	0.932 ± 0.005	**0.816 ± 0.010**	**0.860 ± 0.006**
DeepConv-DTI	0.926 ± 0.008	0.803 ± 0.021	0.851 ± 0.007
SPVec-SGCN-CPI	0.907 ± 0.009	0.741 ± 0.008	0.702 ± 0.009
GraphDTA	0.910 ± 0.019	0.773 ± 0.027	0.708 ± 0.016
GraphformerCPI	0.910 ± 0.003	0.775 ± 0.006	0.826 ± 0.006
DrugBAN	**0.937 ± 0.008**	0.786 ± 0.029	**0.860 ± 0.003**
DSANIB	0.917 ± 0.009	0.780 ± 0.016	0.818 ± 0.012
PocketDTA	0.911 ± 0.007	0.762 ± 0.019	0.670 ± 0.029
FusionDTI	0.920 ± 0.011	0.781 ± 0.024	0.845 ± 0.012
GraphBAN	0.916 ± 0.011	0.774 ± 0.028	0.839 ± 0.011
DESTIN	0.927 ± 0.005	0.803 ± 0.012	0.856 ± 0.006

Detailed analysis of DESTIN and GraphBAN predictions

To investigate the prediction characteristics of DESTIN and GraphBAN, we calculated the numbers of true positives (TPs), true negatives (TNs), false positives (FPs), and false negatives (FNs) for each model. The results are summarized in Supplementary Tables 5-8. Across all datasets, in the inductive scenario, DESTIN and GraphBAN tended to yield a larger number of TNs and TPs, respectively. In contrast, there were no obvious differences between the two methods in the transductive scenario.

Supplementary Table 5: Classification outcomes of DESTIN and GraphBAN on BindingDB under the inductive and transductive scenarios. We used the same random seed to split the dataset as in the previous study (Hadipour et al., 2025).

(A) Inductive scenario

Seed=12	TP	TN	FP	FN
DESTIN	244	649	249	302
GraphBAN	517	176	722	29
Seed=14	TP	TN	FP	FN
DESTIN	284	594	282	344
GraphBAN	600	100	776	28
Seed=16	TP	TN	FP	FN
DESTIN	279	453	293	190
GraphBAN	456	150	596	13
Seed=18	TP	TN	FP	FN
DESTIN	405	553	297	262
GraphBAN	624	110	740	43
Seed=20	TP	TN	FP	FN
DESTIN	249	648	238	470
GraphBAN	687	245	641	32

(B) Transductive scenario

Seed=12	TP	TN	FP	FN
DESTIN	1767	2679	208	266
GraphBAN	1812	2579	308	221
Seed=14	TP	TN	FP	FN
DESTIN	1927	2527	289	177
GraphBAN	1922	2516	300	182
Seed=16	TP	TN	FP	FN
DESTIN	1822	2640	223	235
GraphBAN	1905	2445	418	152
Seed=18	TP	TN	FP	FN
DESTIN	1805	2650	238	227
GraphBAN	1838	2604	284	194
Seed=20	TP	TN	FP	FN
DESTIN	1865	2547	251	257
GraphBAN	1937	2445	353	185

Supplementary Table 6: Classification outcomes of DESTIN and GraphBAN on BioSNAP under the inductive and transductive scenarios. We used the same random seed to split the dataset as in the previous study (Hadipour et al., 2025).

(A) Inductive scenario

Seed=12	TP	TN	FP	FN
DESTIN	282	315	66	126
GraphBAN	321	202	179	87
Seed=14	TP	TN	FP	FN
DESTIN	379	419	101	194
GraphBAN	467	303	217	106
Seed=16	TP	TN	FP	FN
DESTIN	301	312	94	125
GraphBAN	304	248	158	122
Seed=18	TP	TN	FP	FN
DESTIN	273	315	74	131
GraphBAN	281	272	117	123
Seed=20	TP	TN	FP	FN
DESTIN	315	373	78	181
GraphBAN	407	196	255	89

(B) Transductive scenario

Seed=12	TP	TN	FP	FN
DESTIN	1221	1150	226	150
GraphBAN	1213	1039	337	158
Seed=14	TP	TN	FP	FN
DESTIN	1241	1144	212	150
GraphBAN	1177	1146	210	214
Seed=16	TP	TN	FP	FN
DESTIN	1239	1140	211	157
GraphBAN	1169	1135	216	227
Seed=18	TP	TN	FP	FN
DESTIN	1232	1140	247	128
GraphBAN	1145	1130	257	215
Seed=20	TP	TN	FP	FN
DESTIN	1290	1116	189	152
GraphBAN	1232	1054	251	210

Supplementary Table 7: Classification outcomes of DESTIN and GraphBAN on PDBbind2016 under the inductive and transductive scenarios. We used the same random seed to split the dataset as in the previous study (Hadipour et al., 2025).

(A) Inductive scenario

Seed=12	TP	TN	FP	FN
DESTIN	70	69	28	23
GraphBAN	91	11	86	2
Seed=14	TP	TN	FP	FN
DESTIN	52	70	29	48
GraphBAN	90	25	74	10
Seed=16	TP	TN	FP	FN
DESTIN	105	84	52	39
GraphBAN	143	17	119	1
Seed=18	TP	TN	FP	FN
DESTIN	73	49	35	18
GraphBAN	91	0	84	0
Seed=20	TP	TN	FP	FN
DESTIN	92	71	45	31
GraphBAN	122	2	114	1

(B) Transductive scenario

Seed=12	TP	TN	FP	FN
DESTIN	370	277	109	55
GraphBAN	386	111	275	39
Seed=14	TP	TN	FP	FN
DESTIN	359	313	80	59
GraphBAN	374	159	234	44
Seed=16	TP	TN	FP	FN
DESTIN	340	324	94	53
GraphBAN	364	128	290	29
Seed=18	TP	TN	FP	FN
DESTIN	359	302	107	43
GraphBAN	370	125	284	32
Seed=20	TP	TN	FP	FN
DESTIN	355	319	84	53
GraphBAN	356	206	197	52

Supplementary Table 8: Classification outcomes of DESTIN and GraphBAN on KIBA under the inductive and transductive scenarios. We used the same random seed to split the dataset as in the previous study (Hadipour et al., 2025).

(A) Inductive scenario

Seed=12	TP	TN	FP	FN
DESTIN	16	2906	27	926
GraphBAN	887	584	2349	55
Seed=14	TP	TN	FP	FN
DESTIN	65	4075	100	865
GraphBAN	867	679	3496	63
Seed=16	TP	TN	FP	FN
DESTIN	46	1974	101	342
GraphBAN	363	320	1755	25
Seed=18	TP	TN	FP	FN
DESTIN	50	2752	81	476
GraphBAN	400	1463	1370	126
Seed=20	TP	TN	FP	FN
DESTIN	19	2974	23	694
GraphBAN	661	580	2417	52

(B) Transductive scenario

Seed=12	TP	TN	FP	FN
DESTIN	1608	9034	529	655
GraphBAN	2008	7524	2039	255
Seed=14	TP	TN	FP	FN
DESTIN	1568	9089	418	751
GraphBAN	2044	7750	1757	275
Seed=16	TP	TN	FP	FN
DESTIN	1535	9077	452	762
GraphBAN	2017	7011	2518	280
Seed=18	TP	TN	FP	FN
DESTIN	1458	9231	376	761
GraphBAN	1898	7862	1745	321
Seed=20	TP	TN	FP	FN
DESTIN	1423	9142	402	859
GraphBAN	1976	7630	1914	306

Performance comparison of DESTIN, random baseline, and label-permutation control

We compared the performance of DESTIN with that of a random baseline and a label-permutation control. For each test dataset, we calculated the prevalence, corresponding to the AUPR of a random baseline. In the label-permutation control experiment, we randomly shuffled the labels of all samples and then performed model training and performance evaluation using the permuted labels.

Supplementary Table 9 presents the performance metrics of DESTIN, the random baseline, and the label-permutation control across all datasets and data-splitting scenarios. The proposed method consistently outperformed both the random baseline and the label-permutation control. Notably, label permutation resulted in performance comparable to the random baseline. These findings suggest that the proposed method learns genuine relationships between the input features and labels and therefore provides meaningful predictive performance.

Supplementary Table 9: Performance comparison of DESTIN, random baseline, and label-permutation control across all datasets and data-splitting scenarios.

BindingDB: Inductive scenario	AUC	AUPR	F1
DESTIN	0.627 ± 0.033	0.515 ± 0.060	0.694 ± 0.016
Random baseline	0.500 ± 0.000	0.414 ± 0.028	-
Label-permutation control	0.524 ± 0.003	0.432 ± 0.018	0.667 ± 0.001
**BindingDB: Transductive scenario**	**AUC**	**AUPR**	**F1**
DESTIN	0.961 ± 0.005	0.945 ± 0.008	0.904 ± 0.004
Random baseline	0.500 ± 0.000	0.421 ± 0.008	-
Label-permutation control	0.508 ± 0.008	0.428 ± 0.009	0.667 ± 0.000
**BioSNAP: Inductive scenario**	**AUC**	**AUPR**	**F1**
DESTIN	0.806 ± 0.008	0.821 ± 0.014	0.743 ± 0.011
Random baseline	0.500 ± 0.000	0.517 ± 0.006	-
Label-permutation control	0.528 ± 0.010	0.511 ± 0.025	0.667 ± 0.001
**BioSNAP: Transductive scenario**	**AUC**	**AUPR**	**F1**
DESTIN	0.935 ± 0.005	0.932 ± 0.011	0.876 ± 0.004
Random baseline	0.500 ± 0.000	0.507 ± 0.010	-
Label-permutation control	0.494 ± 0.009	0.494 ± 0.009	0.667 ± 0.000
**PDBbind2016: Inductive scenario**	**AUC**	**AUPR**	**F1**
DESTIN	0.721 ± 0.040	0.708 ± 0.059	0.727 ± 0.018
Random baseline	0.500 ± 0.000	0.508 ± 0.011	-
Label-permutation control	0.569 ± 0.020	0.573 ± 0.054	0.672 ± 0.004
**PDBbind2016: Transductive scenario**	**AUC**	**AUPR**	**F1**
DESTIN	0.892 ± 0.012	0.884 ± 0.018	0.832 ± 0.009
Random baseline	0.500 ± 0.000	0.505 ± 0.014	-
Label-permutation control	0.500 ± 0.010	0.497 ± 0.017	0.668 ± 0.001
**KIBA: Inductive scenario**	**AUC**	**AUPR**	**F1**
DESTIN	0.623 ± 0.014	0.274 ± 0.032	0.672 ± 0.003
Random baseline	0.500 ± 0.000	0.186 ± 0.032	-
Label-permutation control	0.527 ± 0.007	0.200 ± 0.001	0.668 ± 0.001
**KIBA: Transductive scenario**	**AUC**	**AUPR**	**F1**
DESTIN	0.927 ± 0.005	0.803 ± 0.012	0.856 ± 0.006
Random baseline	0.500 ± 0.000	0.192 ± 0.003	-
Label-permutation control	0.501 ± 0.003	0.197 ± 0.003	0.667 ± 0.000

Effect of 3D structural information on CPI prediction accuracy at the protein and compound levels

We evaluated how 3D structural information affects the prediction accuracy for individual proteins and compounds. We calculated AUC values at the protein and compound levels and evaluated the differences in AUC distributions among the models using the Wilcoxon signed-rank test. The analysis was performed using compound–protein pairs in the inductive datasets, including PDBbind 2016 (382 proteins and 484 compounds), BindingDB (671 proteins and 2,512 compounds), BioSNAP (694 proteins and 1,265 compounds), and KIBA (187 proteins and 1,042 compounds).

Supplementary Fig. 1(A) shows the AUC distributions at the compound level. For PDBbind 2016, BindingDB, and BioSNAP, DESTIN exhibited significantly higher AUC distributions than GraphBAN ($p < 0.001$). In contrast, no significant difference was observed between the two methods on KIBA.

Supplementary Fig. 1(B) shows the AUC distributions at the protein level. For PDBbind 2016 and BioSNAP, DESTIN exhibited higher AUC distributions than GraphBAN ($p < 0.001$). In contrast, for KIBA, GraphBAN exhibited a significantly higher AUC distribution than DESTIN ($p < 0.05$). No significant differences were observed between the two methods on BindingDB.

These results indicate that 3D structural information for proteins and compounds improves prediction accuracy, particularly for datasets that include structurally diverse molecules.

(A) Compound-level AUC distribution

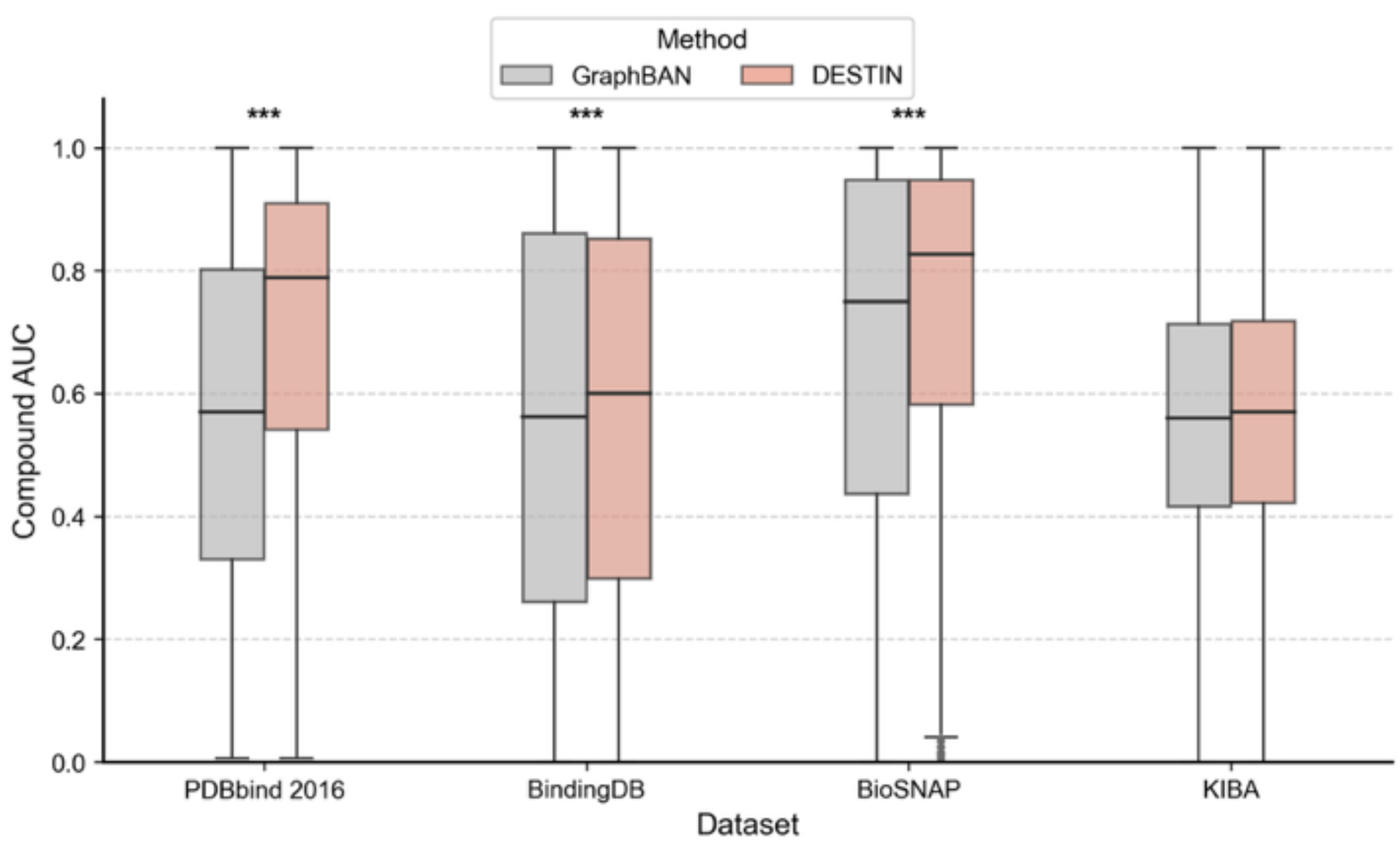

(B) Protein-level AUC distribution

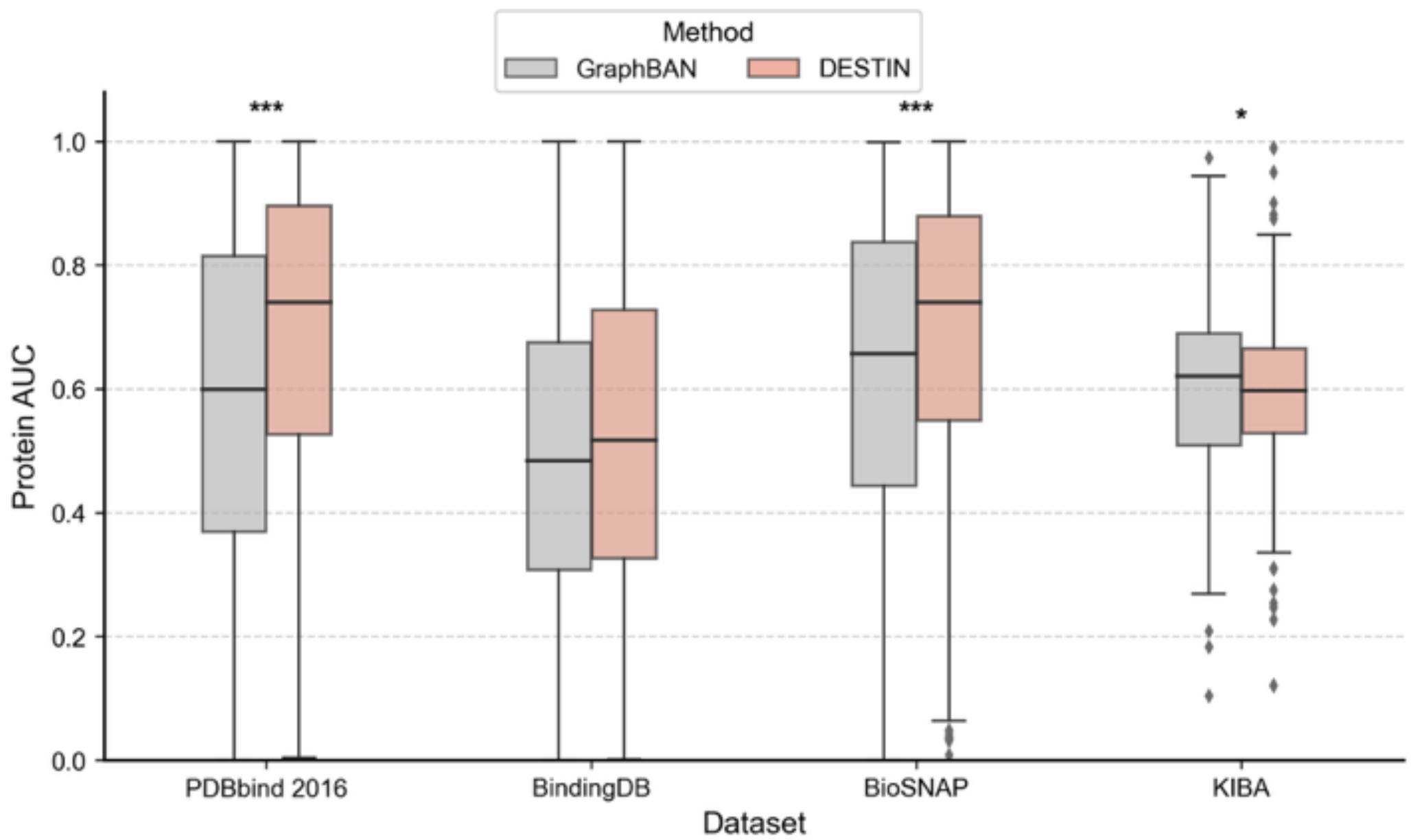

Supplementary Fig. 1: Effect of three-dimensional structural information on CPI prediction performance distributions at the compound and protein levels. AUC distributions calculated at the compound level and the protein level are shown for GraphBAN and DESTIN. (A) Compound-level AUC distributions. (B) Protein-level AUC distributions. The horizontal axis indicates the datasets, and the vertical axis indicates AUC scores for individual compounds or proteins. Each boxplot represents the distribution of mean AUC values for the same compound or protein, averaged over experiments conducted with five different random seeds (12, 14, 16, 18, and 20). Statistical significance was assessed using the Wilcoxon signed-rank test (* $p < 0.05$, *** $p < 0.001$).

Identification of protein categories with improved prediction performance using 3D structural information

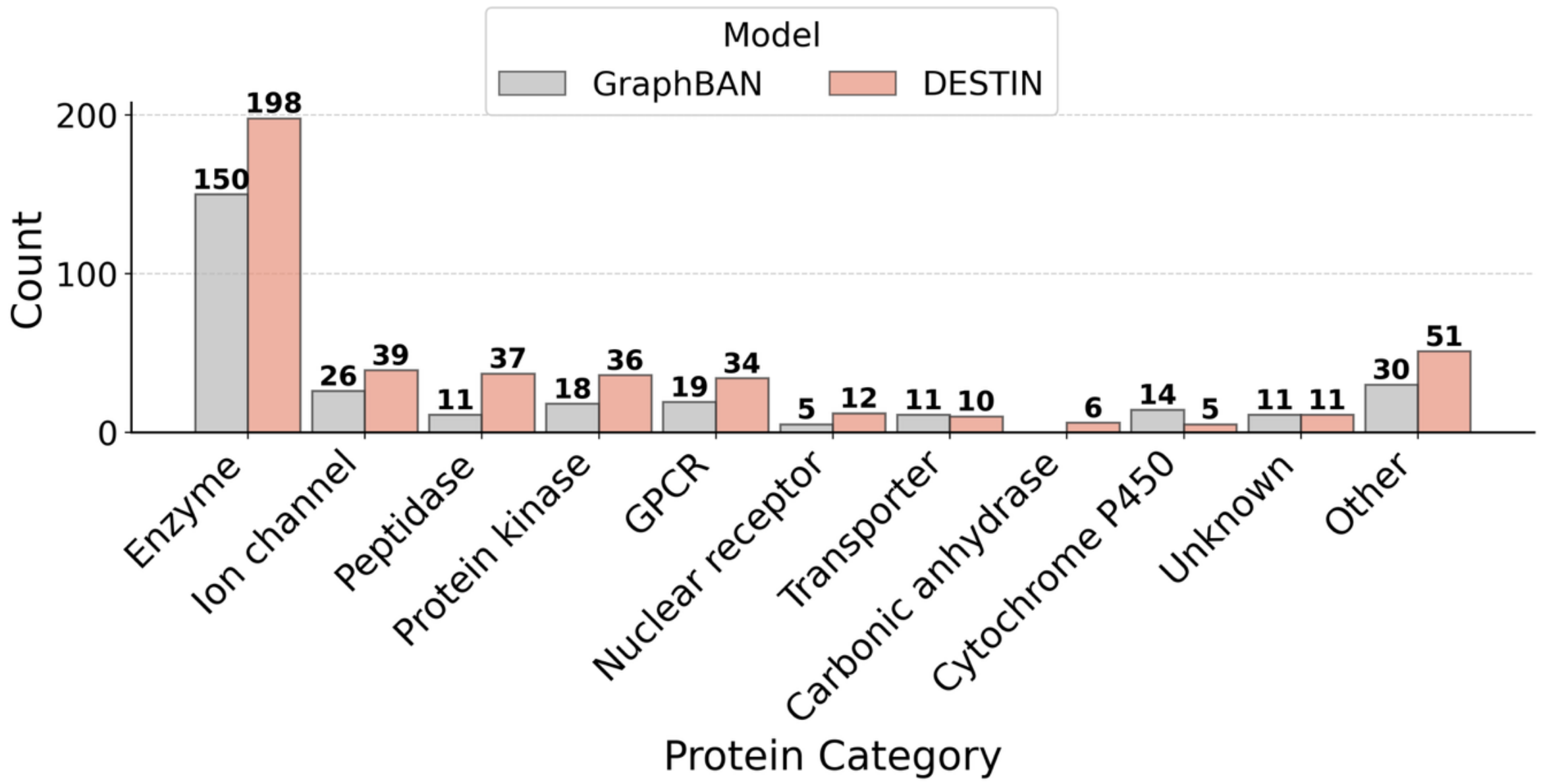

Supplementary Fig. 2: Number of protein categories showing improved prediction performance on BioSNAP. Proteins with protein-level AUC ≥ 0.5 were used for this analysis. Proteins were grouped by UniProt category based on the method that exhibited higher AUC values. The gray bars indicate the number of proteins for which GraphBAN exhibited higher AUC values, and the red bars indicate the number of proteins for which DESTIN exhibited higher AUC values.

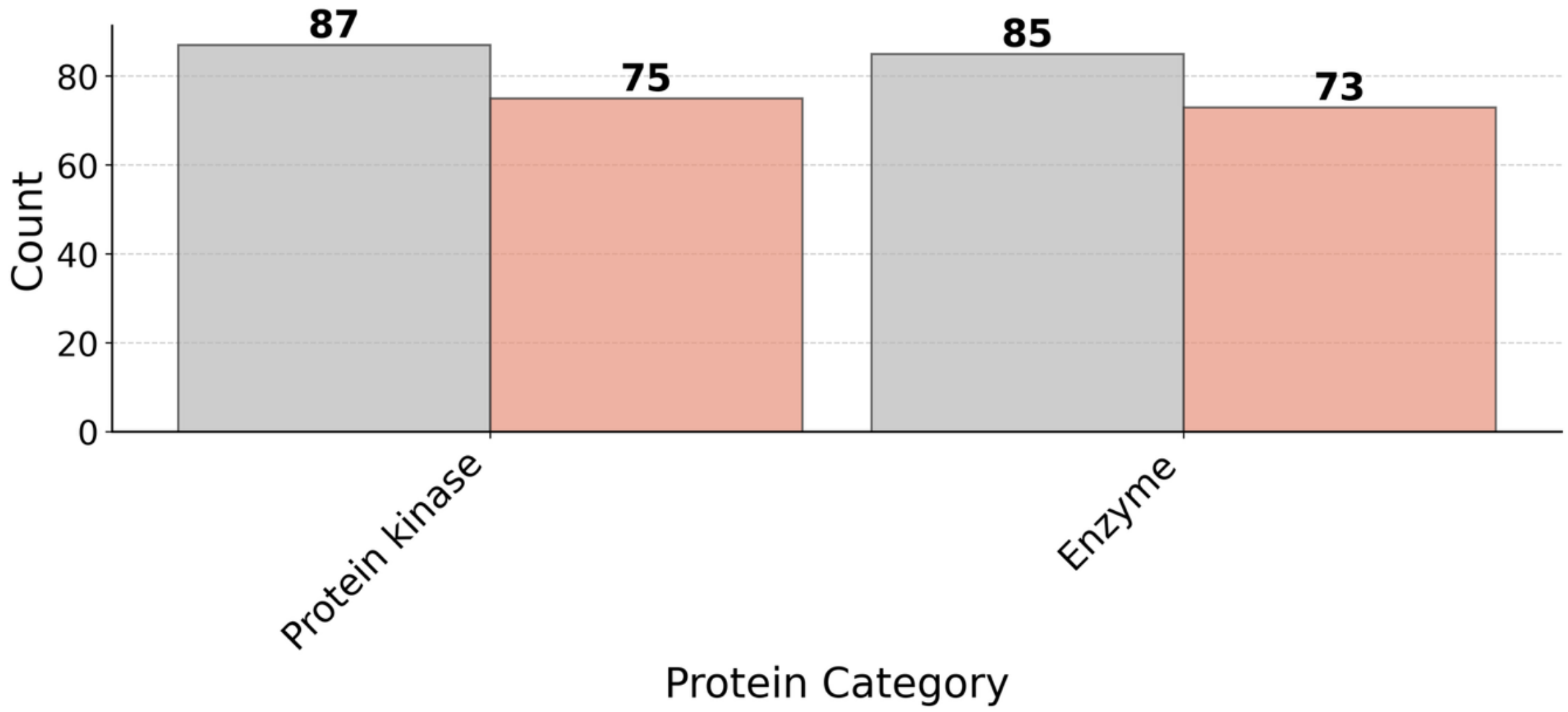

Supplementary Fig. 3: Number of protein categories showing improved prediction performance on KIBA. Proteins with protein-level AUC ≥ 0.5 were used for this analysis. Proteins were grouped by UniProt category based on the method that exhibited higher AUC values. The gray bars indicate the number of proteins for which GraphBAN exhibited higher AUC values, and the red bars indicate the number of proteins for which DESTIN exhibited higher AUC values.

Examples of proteins showing marked differences in predictive performance between models

Protein	Sequence length	GraphBAN AUC	DESTIN AUC	Structure
Nociceptin receptor	370	0.993	0.508	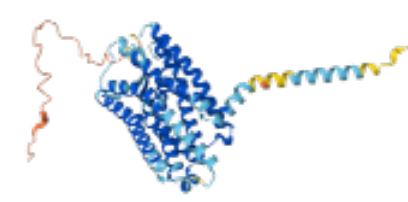
Dihydrofolate reductase	163	0.992	0.452	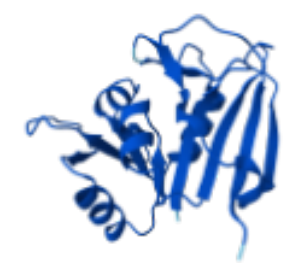
Melanocortin receptor 3	323	0.965	0.416	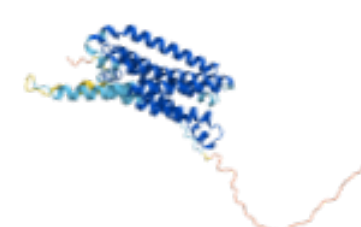
Gamma-aminobutyric acid receptor subunit rho-2	465	0.979	0.597	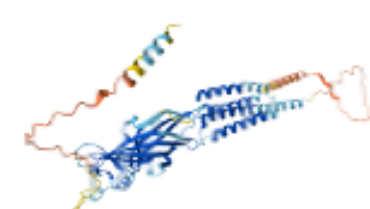
Cyclin-dependent kinase 5 activator 1	307	0.958	0.566	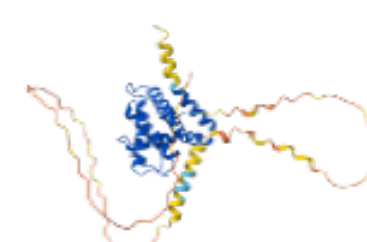
Adenosine receptor A2a	412	0.952	0.587	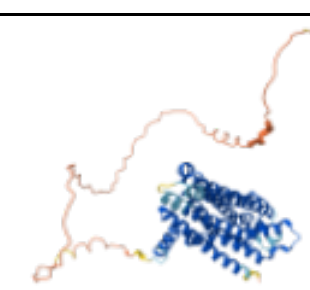
Protein farnesyltransferase/geranylgeranyltransferase type-1 subunit alpha	379	0.934	0.494	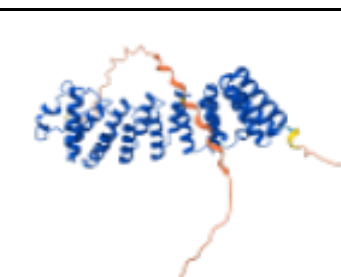

Supplementary Fig. 4: Examples of proteins with pronounced differences in predictive performance between DESTIN and GraphBAN. Proteins for which GraphBAN outperformed DESTIN are shown. In the structures, orange regions indicate residues with low-confidence predictions by AlphaFold2.
